# Distribution of bone tunnel positions and treatment efficacy of bone landmark positioning method for anatomical reconstruction of the anterior cruciate ligament: a case control study

**DOI:** 10.1186/s12891-023-06734-x

**Published:** 2023-07-22

**Authors:** Yan Dong, Yang Gao, Yuanming He, Beixi Bao, Xue Zhao, Peng Cui

**Affiliations:** 1 Department of Orthopedics, Beijing Tongren Hospital, Capital Medical University, Beijing, China; 2 Department of Anesthesiology, Beijing Tongren Hospital, Capital Medical University, Beijing, China

**Keywords:** ACL rupture, Anatomical reconstruction, Bone landmark, Femoral tunnel, Function, Retrospective, Stability

## Abstract

**Background:**

This study aimed to investigate the distribution of femoral tunnel and explore the influences of bone tunnel positions on knee functions. The bone landmark positioning method was used to position the femoral tunnel during the anatomical reconstruction surgery in patients with anterior cruciate ligament (ACL) rupture.

**Methods:**

Data of patients who underwent anatomical reconstruction of the ACL between January 2015 and July 2018, were retrospectively analyzed. The distribution of the femoral tunnel was recorded on 3-D CT after surgery. The tunnel positions were classified into good and poor position groups based on whether the position was in the normal range (24–37% on the *x*-axis and 28–43% on the *y*-axis). The Lysholm and IKDC scores, KT-1000 side-to-side difference, pivot shift test and Lachman test results of the knee joints were recorded, and then the differences in knee joint functions between the two groups were analyzed.

**Results:**

84 eligible patients (84 knees) were finally included in this study. Twenty-two and 62 of the patients were categorized in the good and poor position groups, respectively, and the rate of good position was 26.2%. The distribution of bone tunnel was as follows: (*x*-axis) deep position in 10 patients (12%), normal position in 58 patients (69%), and shallow position in 16 patients (19%); (*y*-axis) high position in 54 patients (64%), normal position in 26 patients (31%), and low position in 4 patients (5%). 1 year later, the Lysholm and IKDC scores were significantly better in the good position group (*P* < 0.05), the KT-1000 side to side difference, the pivot shift test and Lachman test results were better in the good position group (*P* < 0.05).

**Conclusions:**

The bone tunnels were found to be distributed in and beyond the normal range using the bone landmark method to position the femoral tunnel in the single-bundle anatomical reconstruction of ACL, while the rate of good bone tunnel position was low. The knee joint function scores and stability were lower in patients with poor position of the femoral tunnel.

## Background

Anterior cruciate ligament (ACL) is an important structure providing stability for the knee joint. The number of patients with ACL injury has also increased gradually. The rupture of ACL can lead to knee joint instability and pain. Arthroscopic ACL reconstruction is considered as an effective treatment method in patients in whom the conservative treatment has failed [1]. Various factors influence the effects of ACL reconstruction surgery, such as the differences in surgical techniques, types of grafts, diameter of graft, and fixtures. Of these factors, bone tunnel is an important influencing factor. During the surgical procedures of ACL reconstruction, various femoral tunnel positioning methods have already been discovered [2–9]. However, none of the methods are suitable for all the conditions, and no single method can be recommended to treat all types of diseases; instead, suitable methods should be selected according to the lateral femoral wall.

In patients with newly occurred ACL rupture, ligament remnants could generally be found in the femoral footprint region, for which the center of the ligament stump could be selected as the center of the femoral tunnel. The stump-preserving reconstruction could help the healing of ligaments and the preservation of proprioceptors [10, 11]. However, the stump in the femoral footprint region is generally absorbed in patients with old ACL rupture and is difficult to be distinguished, which can make accurate positioning difficult.

Anatomical studies showed several osseous anatomical landmarks at the medial wall of lateral femoral condyle [12], such as the lateral intercondylar ridge (also known as resident’s ridge) and lateral bifurcate ridge. The original footprint region at the femoral side of the ACL is at a rather deep site of the medial side of the lateral femoral condyle, which covers the bifurcate ridge along the resident’s ridge. Purnell et al. suggested that using these bone landmarks could help in accurate positioning of the femoral footprint region, and thus the method was a relatively reliable method [13]. In several studies, the positioning was performed by researchers according to the anatomical landmarks at the lateral femoral wall, and thus the insertion site of ACL was decided [2].

However, after this method was extensively applied in clinical surgeries, various reports were published. Some reports suggested that these bone landmarks might not necessarily be present in all patients, and they might not be clearly distinguished [14–16]. Some other studies reported that the resident’s ridge could be observed in approximately 88% of patients in the case group, while the bifurcate ridge could only be found in approximately 48% of patients [17]. Moloney et al. reported that if depending on bone landmarks only, the femoral positioning site might be deviated by > 2.5 mm from the original footprint center by more than half of surgeons [15]. These findings suggested that the bone landmarks might not always appear, and the selection of the bone tunnel site should not be performed exclusively depending on this positioning landmark.

Previous clinical practices showed that such bone landmarks could not be clearly displayed in surgical procedures in many patients, and some landmarks could not be observed under arthroscopy, influencing the accurate judgment of ACL insertion site by surgeons and leading to misjudgment. The consequent mistakes in the bone tunnel positioning and poor fixation site of grafts could lead to the failure of reconstruction surgeries.

This study aimed to investigate the distribution of the femoral tunnel reconstructed by the conventional positioning method and explore the influences of bone tunnel positions on knee joint functions. The data of all patients who underwent single-bundle anatomical reconstruction of ACL using the method of bone landmark positioning of femoral tunnel were retrospectively analyzed.

## Methods

### Study design and patients

In this retrospective case–control study, the inclusion criteria were as follows: (1) aged 18–50 years; (2) ACL rupture in the knee joint, accompanied by medial or lateral meniscus injury; (3) graft an autogenous hamstring tendon; and (4) patients not treated by single-bundle anatomical reconstruction of ACL.

The exclusion criteria were as follows: (1) aged < 18 years or > 50 years; (2) multiple ligament injuries; (3) lesions in the bilateral knee joints; (4) patients underwent reconstruction using grafts other than the hamstring tendon; and (5) patients underwent double-bundle anatomical reconstruction of ACL.

This study was approved by the ethics committee of the Beijing Tongren Hospital, Capital Medical University. All participants signed the informed consent.

### Data collection

**Fig. 1:**
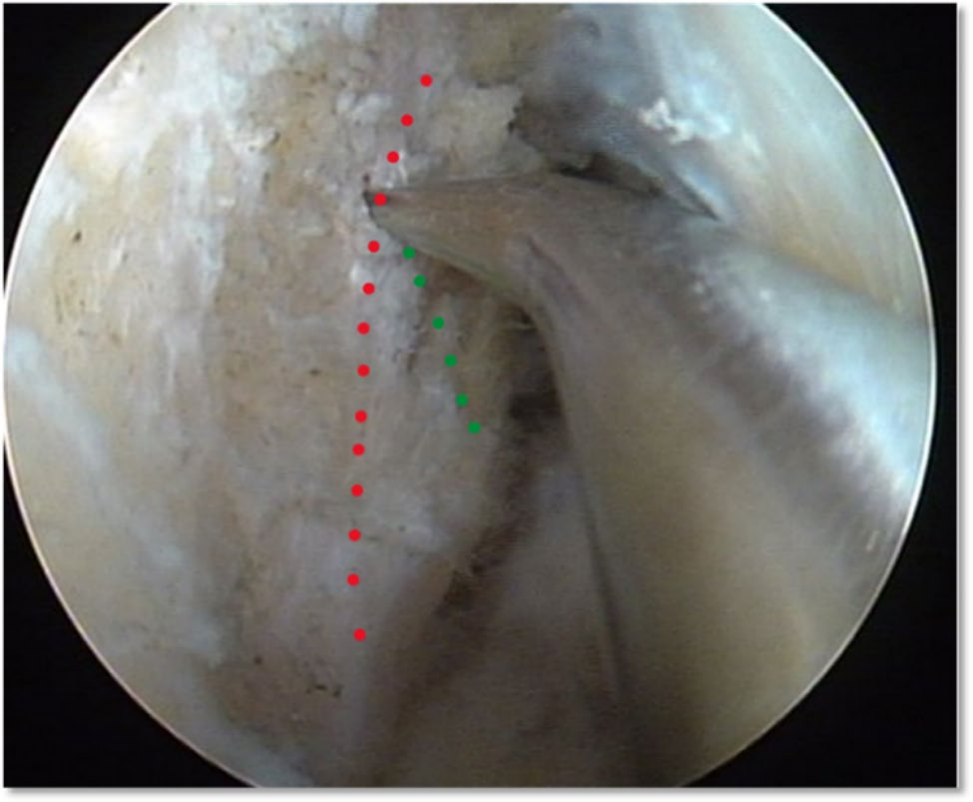
The femoral tunnel was positioned using the method of bone landmark (red dotted line: the resident’s ridge;green dotted line: the bifurcate ridge)

All patients who underwent arthroscopic single-bundle anatomical reconstruction of ACL in the Orthopedics Department of Beijing Tongren Hospital, Capital Medical University, for ACL rupture between January 2015 and July 2018 were included. All surgeries were performed by the same senior surgeon of sports medicine in the Orthopedics Department. All the patients underwent combined anesthesia by general anesthesia plus nerve block. The anteromedial (AM) and anterolateral (AL) portals with the addition of the accessory anteromedial (AAM) portal were used for ACL anatomical reconstruction. ACL reconstruction surgery in the knee joint was performed using the method of bone landmark positioning of femoral tunnel (Fig. 1). If both the resident’s ridge and the bifurcate ridge were displayed, the intersection of the two bony ridges was taken as the localization point; if only the resident’s ridge could be displayed, the midpoint of the bony ridge was selected as the localization point; if only the bifurcate ridge could be displayed, the anterior edge of the ridge was selected as the localization point.

**Fig. 2:**
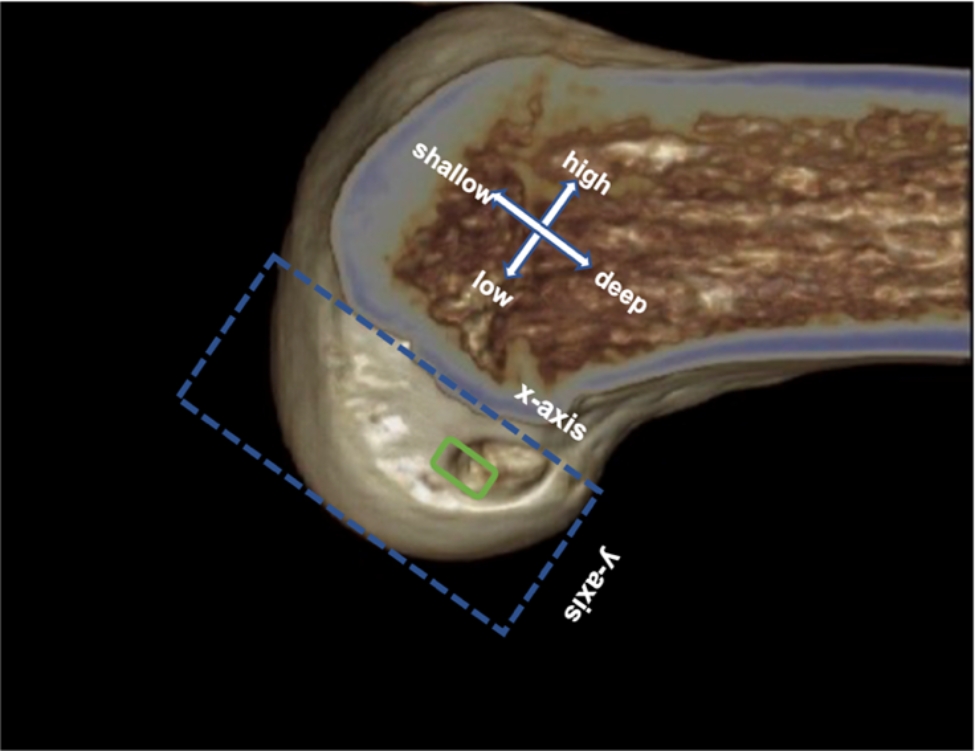
The normal range of positions of bone tunnels at the medial wall of the lateral femoral condyle was 24–37% in the deep–shallow direction (*x*-axis) and 28–43% in the high–low direction (*y*-axis) (green rectangle)

A three-dimensional (3D) computed tomography (CT) examination was performed for the knee joint after the initial surgery, and then the positions of the femoral tunnel were evaluated on CT images. The normal range of positions of bone tunnels at the medial wall of the lateral femoral condyle [18] was 24–37% in the deep–shallow direction (*x*-axis) and 28–43% in the high–low direction (*y*-axis) (Fig. 2). The bone tunnels were categorized into good and poor position groups based on whether the median site of bone tunnel was in the normal range, and the degree of good position was assessed. The good position group included the bone tunnels in 24–37% in the deep–shallow direction (*x*-axis) and in 28–43% in the high–low direction (*y*-axis). The poor position group included the bone tunnels beyond 24–37% in the deep–shallow direction (*x*-axis) or beyond 28–43% in the high–low direction (*y*-axis).

The secondary arthroscopic examinations were performed approximately 1 year after the initial surgery of the patients.

### Efficacy assessment and statistical analysis

#### Assessment of clinical efficacy

The Lysholm and IKDC scores of the knee joints were used to assess the recovery of knee joint functions before the initial ACL reconstruction surgery and secondary arthroscopy exploration. The pivot shift test and the Lachman test were performed under anesthesia to assess the stability of the knee joint. Each patient underwent a KT-1000 arthrometer assessment of anterior tibial translation relative to the femur for laxity of the anterior cruciate ligament.

### Assessment of the bone tunnel position

**Fig. 3:**
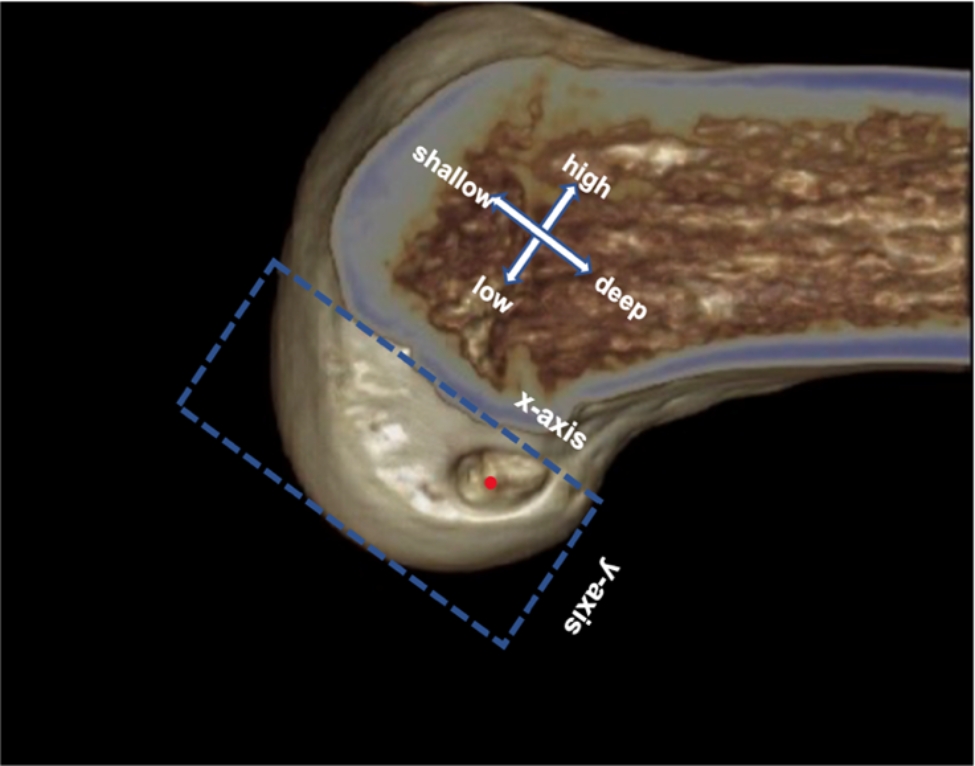
Measurement of the percentage of bone tunnel center (red point) on the *x*-axis and *y*-axis, and assessment of the bone tunnel position

A 3D CT reconstruction of the knee joint was performed after the surgery. The rectangular measuring frame was drawn on the medial view of the lateral femoral condyle (Fig. 3). The percentages of median of the femoral tunnel in the deep–shallow direction (*x*-axis) and high–low direction (*y*-axis) were measured, and the position of the bone tunnel on the medial wall of the lateral femoral condyle of the femur was quantified [19, 20].

### Statistical analysis

The SPSS 22.0 (IBM, NY, USA) software was used for the statistical analysis. The age, body–mass index (BMI), time from injury to initial surgery, position of the femoral tunnel, KT-1000 side to side difference (SSD) and Lysholm and IKDC scores of patients before and after the surgery in the two groups were compared using the *t* test. The sex, side, pivot shift test results, and Lachman test results were compared using the chi-square (*χ*^2^) test. A *P* value < 0.05 indicated a statistically significant difference.

## Results

### Baseline characteristics

In total, data of 101 patients were collected, of which 6 had multiple ligament injuries accompanied by injuries of posterior cruciate ligament or medial collateral ligament, 4 patients underwent ACL reconstruction by allogeneic tendon grafts, and 7 patients lost to follow up. These patients were excluded according to the inclusion and exclusion criteria, and finally 84 eligible patients (84 knees) were included in this study.

**Fig. 4:**
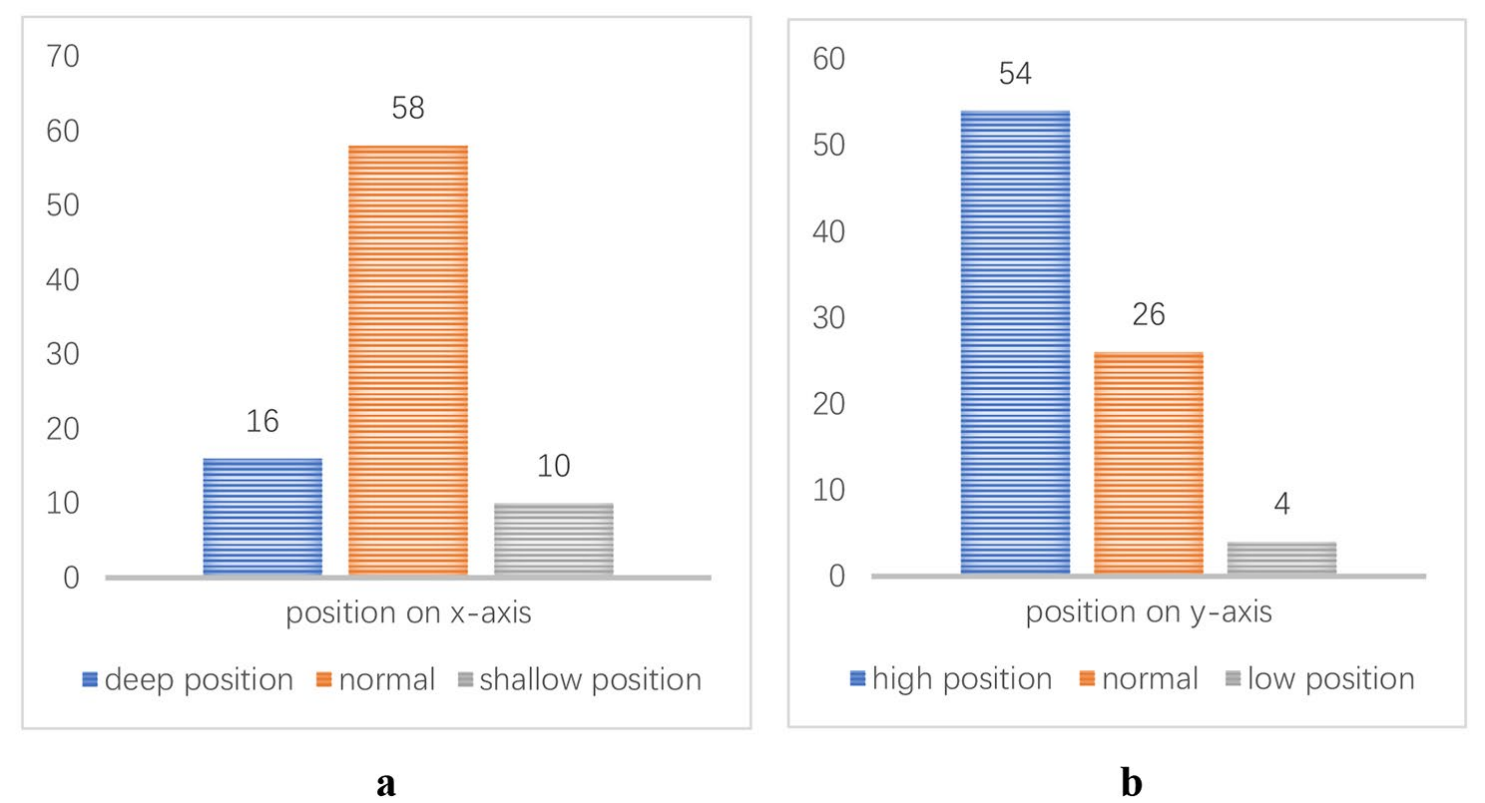
**a** Distribution of bone tunnel positions on the x-axis. **b** Distribution of bone tunnel positions on the y-axis

The normal range of position of the bone tunnel on the medial wall of the lateral femoral condyle was 24–37% in the deep–shallow direction (*x*-axis) and in 28–43% in the high–low direction (*y*-axis) [18]. The distribution of the bone tunnel in the deep–shallow direction (*x*-axis) was as follows (Fig. 4): deep (0–24%) in 10 patients (12%), normal (24–37%) in 58 patients (69%), and shallow (37–100%) in 16 patients (19%). The distribution of bone tunnel in the high–low direction (*y*-axis) was as follows: high (0–28%) in 54 patients (64%), normal (28–43%) in 26 patients (31%), and low (43–100%) in 4 patients (5%).

The patients were categorized into good and poor position groups based on whether the femoral tunnel center was in the normal range. Finally, 22 patients, including 16 males and 6 females, were in the good position group. Specifically, the lesion was in the left knee and right knee in 12 and 10 patients, respectively. The BMI of the patients was 27.1 ± 4.2, and the position of the tibial tunnel (anterior–posterior) was 40.8% ± 3.2%. Sixty-two patients, including 48 males and 14 females, were in the poor position group. Specifically, the lesion was in the left knee and right knee in 40 and 22 patients, respectively. The BMI of the patients was 28.2 ± 3.6, and the position of the tibial tunnel (anterior–posterior) was 42.1% ± 4.3%. The rate of good bone tunnel position was 26.2%. The age, sex, side, and BMI were not significantly different between the two groups (*P* > 0.05) (Table 1).

**Table 1:** Baseline data of patients in the two groups

	Good position group (*n* = 22)	Poor position group (*n* = 62)	*P* value
Mean age (year)	29.8 ± 8.5	32.3 ± 9.4	0.28
Male (*n*)	16	48	0.66
Female (*n*)	6	14	
Left knee (*n*)	12	40	0.41
Right knee (*n*)	10	22	
BMI	27.1 ± 4.2	28.2 ± 3.6	0.24
Position of the tibial tunnel (anterior–posterior) (%)	40.8 ± 3.2	42.1 ± 4.3	0.20

### Comparison of Lysholm and IKDC scores of the knee joint

The knee joint scores of patients before the secondary arthroscopy exploration were compared, and the Lysholm and IKDC scores are shown in Tables 2 and 3.

The mean Lysholm score of the knee joint increased from 41.5 ± 7.9 (before the reconstruction surgery) to 85.2 ± 5.5 (after the reconstruction surgery) in the good position group and from 42.1 ± 10.6 (before the reconstruction surgery) to 81.2 ± 6.8 (after the reconstruction surgery) in the poor position group. The statistical analysis showed that the postoperative score was significantly different between the two groups (*P* = 0.02). The mean IKDC score of the knee joint increased from 42.8 ± 8.2 (before the reconstruction surgery) to 84.3 ± 5.7 (after the reconstruction surgery) in the good position group and from 44.8 ± 10.1 (before the reconstruction surgery) to 79.9 ± 6.4 (after the reconstruction surgery) in the poor position group. The statistical analysis showed that the postoperative score was significantly different between the two groups (*P* = 0.0056).

### Comparison of the knee joint stability

The findings of the knee joint stability are shown in Tables 2 and 3.

The pivot shift test of the knee joint was performed before the initial ACL reconstruction, which showed that the stability was of grade 0, 1, 2, and 3 in 0, 16, 6, and 0 patients in the good position group and in 0, 44, 18, and 0 patients in the poor position group, respectively. The statistical analysis showed that the results were not significantly different between the two groups (*P* = 0.88). The Lachman test showed that the stability was of grade 1, 2, and 3 in 2, 16, and 4 patients in the good position group and in 4, 48, and 10 patients in the poor position group, respectively. The statistical analysis showed that the results were not significantly different between the two groups (*P* = 0.98).

**Table 2:** Comparison of function scores and stability of the knee joint between the two groups before the initial ACL reconstruction surgery

	Good position group (*n* = 22)	Poor position group (*n* = 62)	*P* value
Lysholm (point)	41.5 ± 7.9	42.1 ± 10.6	0.81
IKDC (point)	42.8 ± 8.2	44.8 ± 10.1	0.41
Pivot shift test (grade 0, 1, 2, and 3; *n*)	0/16/6/0	0/44/18/0	0.88
Lachman test (grade 1, 2, and 3; *n*)	2/16/4	4/48/10	0.98
KT-1000 side-to-side difference (mm)	7.7 ± 2.5	7.2 ± 2.8	0.46

The pivot shift test of the knee joint after the initial ACL reconstruction (before the secondary arthroscopy exploration) showed that the stability was of grade 0, 1, 2, and 3 in 16, 6, 0, and 0 patients in the good position group and in 20, 42, 0, and 0 patients in the poor position group, respectively. The statistical analysis showed that the results were significantly different between the two groups (*P* = 0.0011). The Lachman test showed that the stability was of grade 1, 2, and 3 in 18, 4, and 0 patients in the good position group and in 28, 28, and 6 patients in the good position group, respectively. The statistical analysis showed that the results were significantly different between the two groups (*P* = 0.0026). The statistical analysis showed that the results of KT-1000 side-to-side difference were significantly different between the two groups (*P* = 0.0014).

**Table 3:** Comparison of function scores and stability of the knee joint between the two groups after the initial ACL reconstruction surgery

	Good position group (*n* = 22)	Poor position group (*n* = 62)	*P* value
Lysholm (point)	85.2 ± 5.5	81.2 ± 6.8	0.02
IKDC (point)	84.3 ± 5.7	79.9 ± 6.4	0.0056
Pivot shift test (grade 0, 1, 2, and 3; *n*)	16/6/0/0	20/42/0/0	0.0011
Lachman test (grade 1, 2, and 3; *n*)	18/4/0	28/28/6	0.0026
KT-1000 side-to-side difference (mm)	2.2 ± 1.7	4.1 ± 2.5	0.0014

### Arthroscopy exploration findings 1 year after initial ACL reconstruction

#### Continuity of the graft

**Fig. 5:**
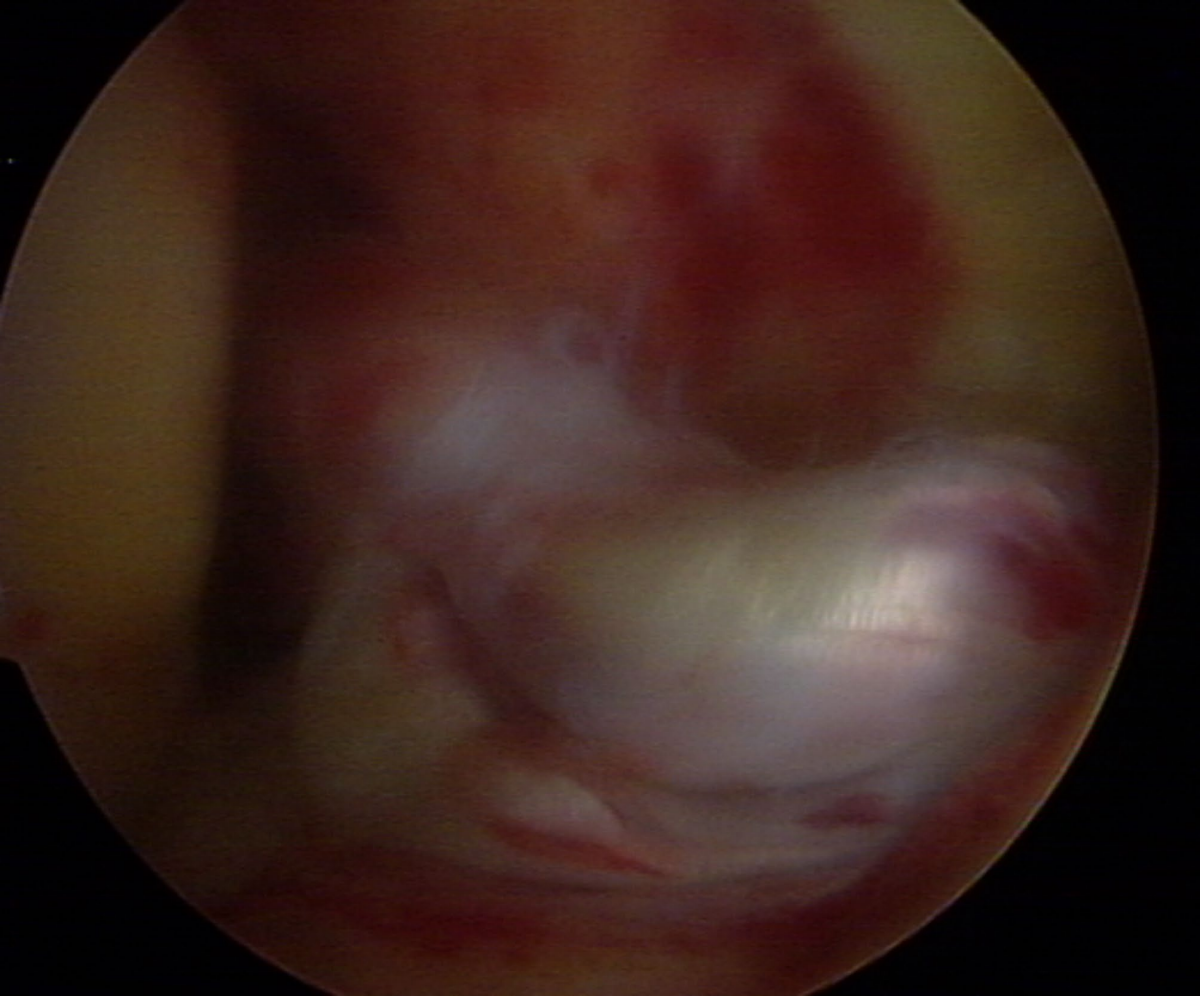
Secondary arthroscopy exploration showing graft loosening and complete rupture

The graft for ACL reconstruction in the knee joints of three patients in the poor position group was completely ruptured (Table 4; Fig. 5). Both patients with the graft completely ruptured underwent revision reconstruction of ACL. The exploration showed partial tearing of the ACL graft in nine patients, all of which were in the poor position group, and the sites of tearing were mainly at distal one third of the graft that inclined to the lateral side, especially at the site where the graft contacted the fossa intercondyloidea during the flexion and extension of knee joint.

### Tension of grafts

**Fig. 6:**
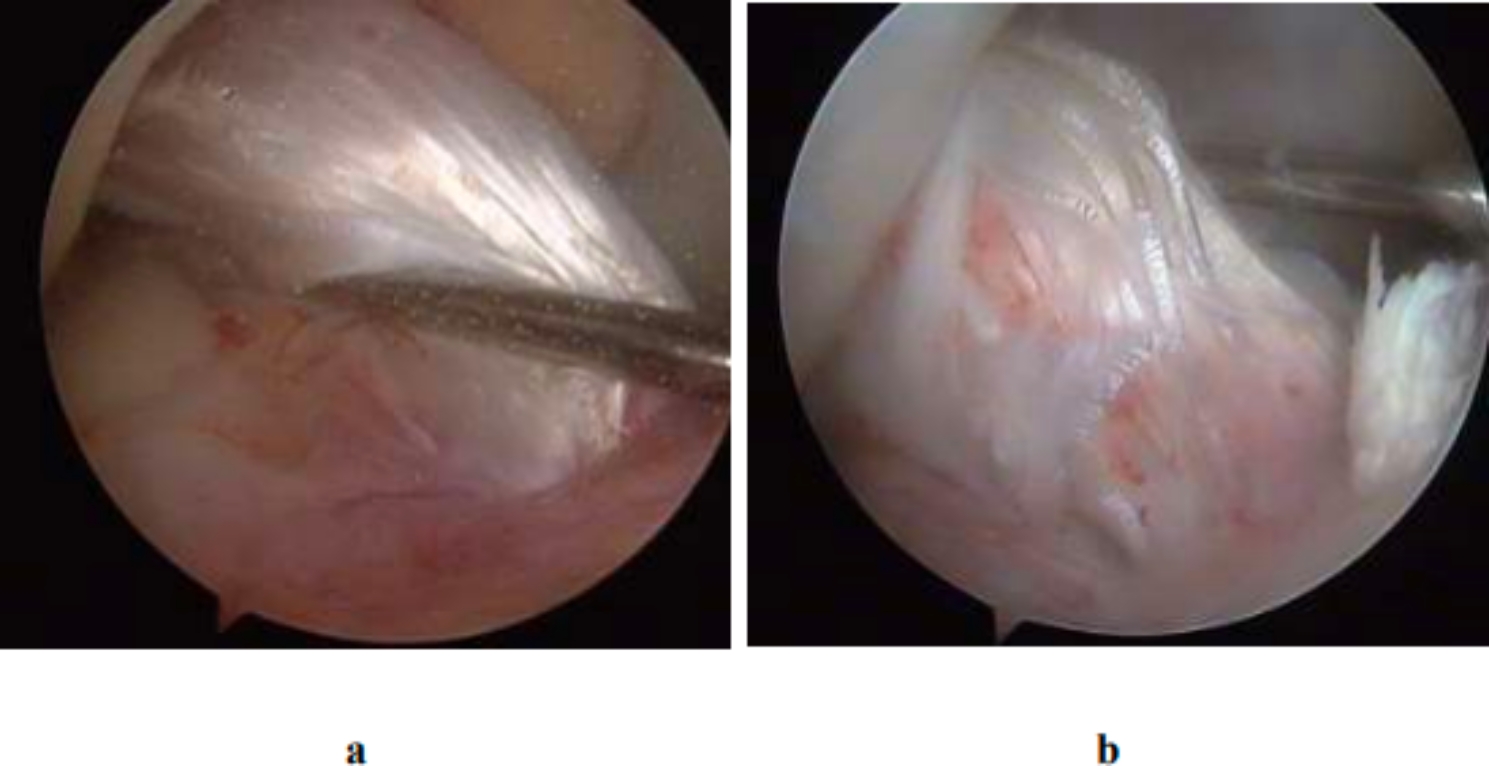
**a** Graft with high tension. **b** Graft with mild loosening

For measuring the tension of grafts, the knee joint was required to bend 30°. Then, a probing hook was used to clasp the major part of the graft from behind, and then pulled forward to assess the tension of the graft (Fig. 6). If the graft had high tension and showed the feeling of hard insertion, the tension recovery was considered good, which was found in 15 patients (68%) in the good position group and 34 patients (55%) in the poor position group. If the graft was loosened forward but still showed hard insertion at the end point, the graft was considered with slight loosening, which was found in 7 patients (32%) in the good position group and 25 patients (40%) in the poor position group. If the graft was completely loosened and showed no feeling of insertion, the graft was considered ruptured, which was found in 3 patients (5%) in the poor position group.

### Coverage of synovial membrane on the surface of the graft

**Fig. 7:**
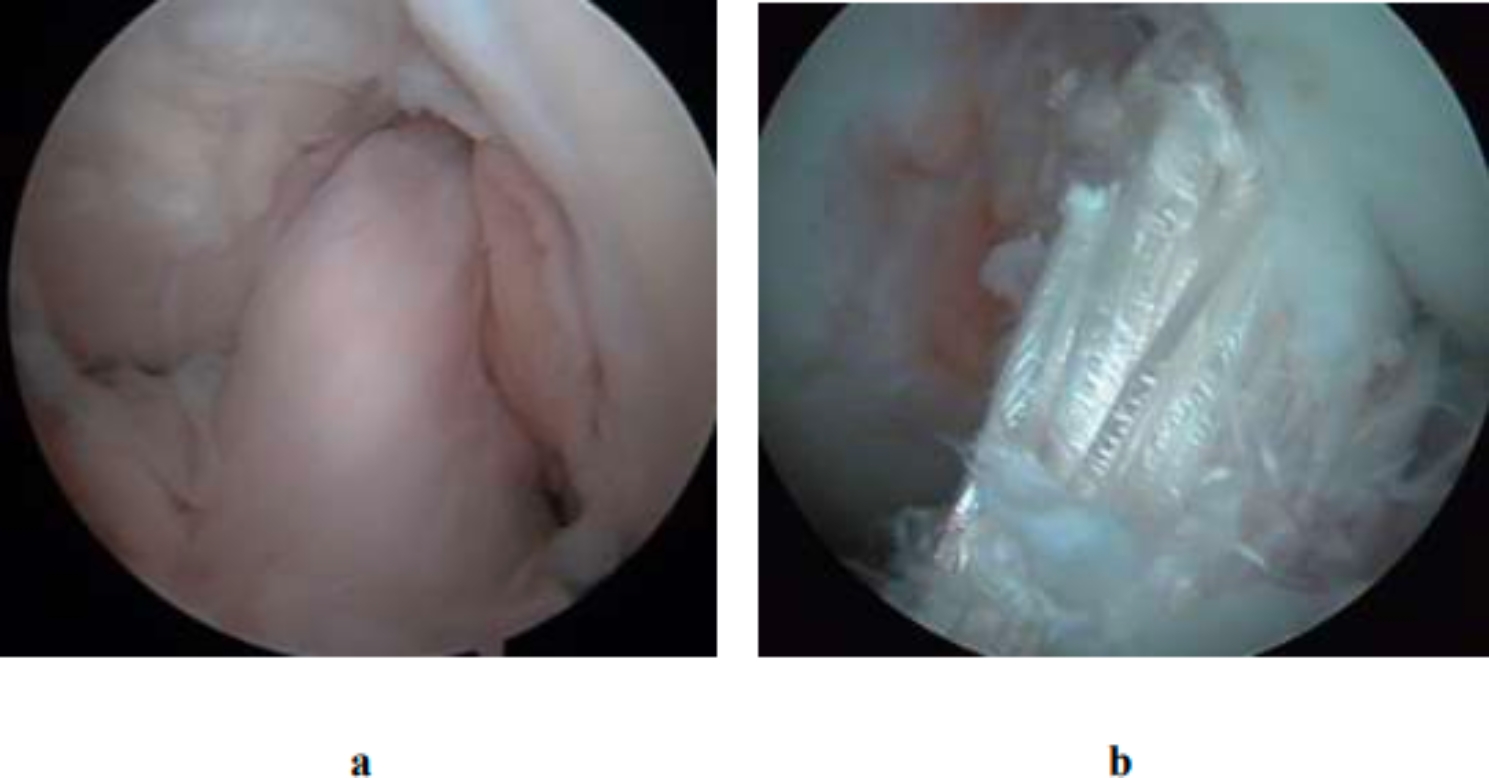
**a** Good synovial membrane coverage. **b** Poor synovial membrane coverage

The coverage rate of synovial membrane on the surface of graft ≥ 50% was considered good coverage, which was found in 15 knees (68%) in the good position group and in 35 knees (56%) in the poor position group (Fig. 7). The coverage rate of the synovial membrane on the surface of graft < 50% was considered poor coverage, which was found in 7 knees (32%) in the good position group and in 27 knees (44%) in the poor position group. The position of poor synovial membrane coverage on the ligament graft was mainly anterior to the graft.

### Meniscus tearing

Meniscus tearing was found in 30 patients at the initial ACL reconstruction, including medial meniscus tearing in 10 patients, lateral meniscus tearing in 14 patients, and medial and lateral meniscus tearing in 6 patients. During the surgery of initial ACL reconstruction, 14 patients also underwent meniscus suturing and 16 patients also underwent meniscus plasty. The secondary surgery of arthroscopy exploration showed that all who underwent meniscus suturing recovered. The secondary exploration showed new meniscus tearing in 3 patients, which occurred at the meniscus free edge (white–white zone) and was treated by partial meniscectomy and plasty.

### Cartilage injury

The secondary arthroscopy exploration showed cartilage injury at the femoral trochlea in 19 patients (9 patients in the good position group and 10 patients in the poor position group), with grade 2 injuries; cartilage injury on the surface of the patellar joint in 8 patients (4 patients each in the good and poor position groups), with grade 2 injuries; and cartilage injury at the medial femoral condyle in 6 patients (3 patients in the good position group, and 3 patient in the poor position group), with grade 2 injuries.

### Complications

**Fig. 8:**
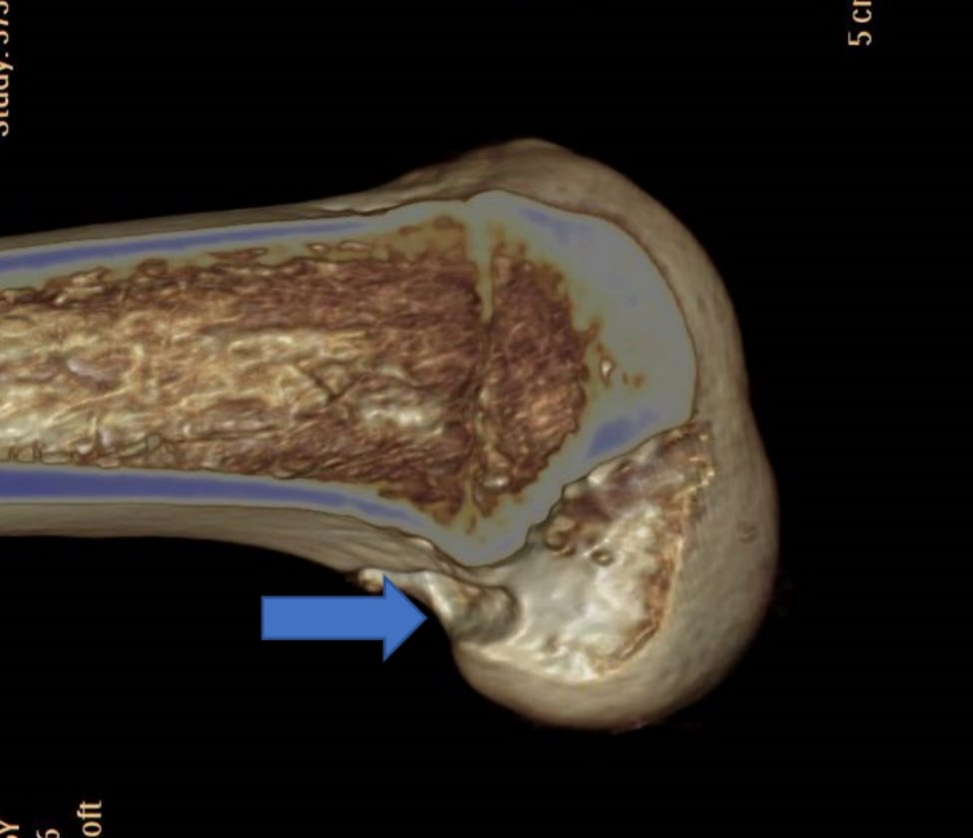
Rupture of the posterior wall of the femoral tunnel during ACL reconstruction surgery

No infection was found in patients after the surgery (Table 4). Posterior wall rupture of the femoral tunnel occurred in 2 patients in the poor position group during the surgery (Fig. 8). On removing the internal fixation 1 year after the surgery, arthroscopy exploration confirmed complete rupture of the ligament graft in 3 patients in the poor position group, and they underwent revision reconstruction of the ACL. Postoperative pain in the knee joint was found in 3 patients, with a visual analog scale score of 3 points, and no specific treatment was required. Re-tearing of meniscus was found in 3 patients, in which no significant symptom was found. Synarthrophysis in the knee joint was found after the surgery in one patient (extension: 0°, and flexion 90°), and the.

**Table 4:** Secondary arthroscopy exploration findings and complications

	Good position group (*n* = 22)	Poor position group (*n* = 62)
Continuity of graft (good/ruptured; *n*)	22/0	50/12 (complete rupture in 3 patients, and partial rupture in 9 patients)
Tension of graft (high/mild loosening/complete loosening; *n*)	15/7/0	34/25/3
Synovial membrane coverage (good/poor; *n*)	15/7	35/27
Re-tearing of the meniscus (*n*)	1	2
Cartilage injury (femoral trochlear/patellar joint surface/medial femoral condyle; *n*)	9/4/3	10/4/3
Bone wall rupture (*n*)	0	2
Internal fixation stimulation (*n*)	1	0
Synarthrophysis (*n*)	0	1

patient was treated by surgical release. Stimulation of the skin by the tail of the extrusion screw on the tibial side was found in one patient, which was corrected by surgery, and then the symptoms were alleviated.

## Discussion

The findings of this study showed that when using conventional methods, such as bone landmarks, for positioning the femoral tunnel in ACL reconstruction, the position of the bone tunnel could easily deviate from the normal range, and the accurate rate of bone tunnel position was low. The function scores and stability of the knee joint were both lower in patients with poor femoral tunnel positions compared with those with good femoral tunnel positions.

Parkar showed that the normal range of the bone tunnel of ACL was 24–37% in the deep–shallow direction (*x*-axis) and 28–43% in the high–low direction (*y*-axis) [18]. In this study, the bone landmark positioning method was the major method for positioning the femoral tunnel. The analysis of positions of the femoral tunnel showed that the femoral tunnels did not concentrate in the normal range of the bone tunnel, but tended to show the diffused distribution in the high–low and deep–shallow directions on the medial wall of the lateral femoral condyle. The patients were categorized into good and position groups based on whether the center of the bone tunnel was in the normal range. Finally, 22 patients were in the good position group, 62 patients were in the poor position group, and the rate of good bone tunnel position was only 26.2%. Moloney et al. showed that if only depending on bone landmarks, the femoral positioning site might be deviated by > 2.5 mm from the original footprint center by more than half of surgeons, indicating that the positioning by intra-articular bone landmarks was not reliable [9, 15, 21]. The findings were in agreement with the results of the present study, which showed that the positions of femoral tunnels were not concentrated, and the rate of good bone tunnel position was low. Therefore, although the conventional positioning method was classic, the accuracy rate was not high in clinical practices due to the diffusion of the bone tunnel, and thus the method could not be acknowledged and accepted by all surgeons in performing ACL reconstruction. Indeed, the experience and skill level of different surgeons can affect the accuracy of the positioning. To eliminate this variation, all our surgeries were performed by the same senior surgeon.

Various attempts have been made by researchers to explore the reference sites for positioning the femoral tunnel during ACL reconstruction. For instance, Andreas Weiler et al. suggested that the posterior horn of the lateral meniscus could be used as a reference site for positioning the femoral tunnel [22]. Several investigators also used self-designed drilling directors to help in the positioning and drilling of the femoral tunnel [23–26]. In previous studies, researchers used the posterior apex of the deep cartilage of the lateral femoral condyle as a reference and a ruler for intraoperative measuring [27], achieving good positioning effects.

The pivot shift test in this study after ACL reconstruction surgery showed that 32.3% (20/62) and 72.7% (16/22) of patients in the poor and good position groups were with grade 0 stability, respectively. The Lachman test showed that 45.2% (28/62) and 81.8% (18/22) of patients in the poor and good position groups were with grade 1 stability, respectively. The findings of both pivot shift test and Lachman test indicated that the stability of the knee joint was lower in the poor position group. Placing the bone tunnel at different sites led to different length variations of the graft during the flexion and extension of the knee joint. The findings of tests showed that when placing the bone tunnel at a relatively high position in the footprint region, the length change of grafts during the flexion and extension of the knee joint ranged 1–4 mm. When placing the bone tunnel at the center of the footprint region, the length change of grafts during the flexion and extension of the knee joint ranged 5–7 mm [28]. While placing the bone tunnel at a relatively low position in the footprint region that was closer to the lateral cord region, the length changes of grafts during the flexion and extension of the knee joint were as long as 1 cm. When fixing graft in the isometric region, graft loosening or poor control of forward stability of the knee joint, or restriction of the knee joint extension could occur during large-angle flexion and extension, which could even lead to graft rupture and failure [29]. These findings demonstrated that when the position of the femoral tunnel was poor, such as too low, the length change of the graft during flexion and extension of the knee joint was too long and thus the tension was too high, which could even induce the rupture of the graft [30]. When the bone tunnel position was poor, the nonisometry during flexion and extension of the knee joint could induce the passive stretching of the graft, leading to joint loosening and stability reduction. If the position of the femoral tunnel of the graft was too high, the angle of the graft was too vertical and thus influenced the stability of the knee joint. The findings of this study demonstrated that the stability of the knee joint was higher in the good position group than in the poor position group. However, the stability of the knee joint in the good position group still did not achieve 100% recovery (72.7%, 81.8%), indicating even placing of the graft in the footprint region. If the position was too distant from the near-isometric point in the footprint region, the low position in the footprint region could induce the length change of the graft due to nonisometry of the graft during flexion and extension of the knee joint, and thus influenced the recovery of the knee joint stability.

The secondary arthroscopy exploration 1 year after the surgery showed partial tearing of the ACL graft in some patients, and the site of tearing was mainly at distal one third of the graft that inclined to the lateral side. We speculated that the insertion site of the graft on the femoral side was too high, and the insertion site on the tibial side was too shallow. In addition, the postoperative hyperplasia and stenosis in the intercondylar fossa leading to the impact of intercondylar fossa against the lower insertion site of the graft during flexion and extension of the knee joint could also be associated with tearing. The long-term repeated impact stimulations could lead to the tearing of graft at lower insertion. The region of the ACL graft not covered by the synovial membrane was mainly at the front part of the graft, which could be associated with the repeated impact and friction of the graft by the intercondylar fossa during flexion and extension of the knee joint. This phenomenon was observed in both groups. Therefore, further studies are needed to investigate whether it is also associated with the morphology of the intercondylar fossa in addition to the bone tunnel position.

This study had several limitations: [1] This was a retrospective study and the sample size of this study was relatively small, so it may lead to biases and limitations in interpreting the results;[2]the subjective perceptions of evaluators could influence the assessment of stability grades when using the pivot shift test and Lachman test to assess the joint stability, which could be more objective if relevant data could be used to reflect the stability degree of the joint; and [3] various factors influenced the joint function, while the position of the bone tunnel was only one of the factors. More studies are needed to further explore the other factors related to the functions.

## Conclusions

When using the bone landmark method to position the femoral tunnel in the single-bundle anatomical reconstruction of ACL, the bone tunnels were found to be distributed in and beyond the normal range, while the rate of good bone tunnel position was low. The knee joint function scores and stability were lower in patients with poor position of the femoral tunnel than in those with good position of the femoral tunnel. Therefore, more precise positioning methods for femoral tunnel are needed to help restore better knee joint functions and guarantee the success of reconstruction surgery, based on which the positioning site could be placed in the normal range when selecting the femoral tunnel position in ACL reconstruction.

## Data Availability

The datasets generated during the current study are not publicly available due to another article containing these data has not yet available published but are available from the corresponding author on reasonable request.
